# Cornelia de Lange Syndrome as Paradigm of Chromatinopathies

**DOI:** 10.3389/fnins.2021.774950

**Published:** 2021-11-05

**Authors:** Ilaria Parenti, Frank J. Kaiser

**Affiliations:** ^1^Institut für Humangenetik, Universitätsklinikum Essen, Universität Duisburg-Essen, Essen, Germany; ^2^Essener Zentrum für Seltene Erkrankungen (EZSE), Universitätsklinikum Essen, Essen, Germany

**Keywords:** Cornelia de Lange syndrome (CdLS), chromatinopathies, transcriptional regulators, chromatin remodelers, cohesin

## Abstract

Chromatinopathies can be defined as a class of neurodevelopmental disorders caused by mutations affecting proteins responsible for chromatin remodeling and transcriptional regulation. The resulting dysregulation of gene expression favors the onset of a series of clinical features such as developmental delay, intellectual disability, facial dysmorphism, and behavioral disturbances. Cornelia de Lange syndrome (CdLS) is a prime example of a chromatinopathy. It is caused by mutations affecting subunits or regulators of the cohesin complex, a multisubunit protein complex involved in various molecular mechanisms such as sister chromatid cohesion, transcriptional regulation and formation of topologically associated domains. However, disease-causing variants in non-cohesin genes with overlapping functions have also been described in association with CdLS. Notably, the majority of these genes had been previously found responsible for distinct neurodevelopmental disorders that also fall within the category of chromatinopathies and are frequently considered as differential diagnosis for CdLS. In this review, we provide a systematic overview of the current literature to summarize all mutations in non-cohesin genes identified in association with CdLS phenotypes and discuss about the interconnection of proteins belonging to the chromatinopathies network.

## Introduction

Cornelia de Lange syndrome (CdLS, OMIM # 122470, #300590, #610759, #614701, and #300882) is a multisystem developmental disorder named after the Dutch pediatrician Cornelia de Lange, who reported in 1933 two unrelated patients with comparable features. Nowadays, its prevalence is estimated between 1/10,000 and 1/30,000 live births (Kline et al., 2007). A distinct craniofacial appearance, pre- and post-natal growth retardation, intellectual disability, developmental delay, behavioral issues, and limb anomalies are the main clinical features of CdLS, albeit observed with variable expressivity (Kline et al., 2018). The first international consensus statement for CdLS has recently introduced a scoring system to classify the severity of the syndrome and help select the most appropriate pipeline for genetic testing. A score ≥11 confirms the clinical diagnosis of CdLS also in the absence of a molecular diagnosis (Kline et al., 2018).

The genetic etiology of CdLS is mainly attributable to variants affecting the function of the deeply conserved protein complex known as cohesin (Kline et al., 2018). Variants in the cohesin regulator NIPBL are the most frequent cause of CdLS and account for approximately 70% of cases. Other subunits or regulators of the complex (SMC1A, SMC3, RAD21, and HDAC8) are responsible altogether for 10–15% of cases (Kline et al., 2018). Variants in additional cohesin-associated proteins like MAU2, STAG1, and STAG2 have been associated with CdLS or phenotypes reminiscent of CdLS in few individuals (Lehalle et al., 2017; Mullegama et al., 2017; Soardi et al., 2017; Yuan et al., 2019; Parenti et al., 2020).

The cohesin complex performs numerous functions that are essential for cell survival, including sister chromatid cohesion, DNA repair, maintenance of genomic stability, transcriptional regulation, and chromatin regulation by mediating long-range interactions between distant genomic regions and contributing to the formation of topologically associating domains (Zhu and Wang, 2019). Sister chromatid cohesion is the best-characterized role of the complex. However, cell lines of individuals with CdLS do not display cohesion defects (Castronovo et al., 2009). A global dysregulation of gene expression is instead observed in these cells (Liu et al., 2009; Izumi et al., 2015; Yuan et al., 2015).

Hence, an altered functionality of the cohesin complex in the context of transcriptional regulation and chromatin remodeling rather than sister chromatid cohesion can be held accountable for the onset of the disease phenotype (Yuan et al., 2015). In line with these findings, several patients with CdLS were found to carry variants in regulators of gene expression and chromatin architecture other than cohesin. Notably, the majority of these genes have been previously associated with neurodevelopmental disorders sharing a partial phenotypical overlap with CdLS, such as Rubinstein-Taybi syndrome (RSTS, OMIM #180849), KBG syndrome (KBGS, OMIM #148050), Coffin-Siris syndrome (CSS, OMIM #135900), or Wiedemann-Steiner syndrome (WDSTS, OMIM #605130) (Petrif et al., 1995; Roelfsema et al., 2005; Sirmaci et al., 2011; Jones et al., 2012; Tsurusaki et al., 2012; Asadollahi et al., 2013; Grozeva et al., 2014; Hamdan et al., 2014; Hao et al., 2015; O’Rawe et al., 2015; Olley et al., 2018). Not surprisingly, the aforementioned disorders are often considered as a differential diagnosis for CdLS. On the other hand, variants in cohesin genes have been identified in individuals with neurodevelopmental disorders other than CdLS, such as CSS, WDSTS, Rett-like syndrome, or syndromic intellectual disability (Harakalova et al., 2012; Tzschach et al., 2015; Yuan et al., 2015; Yuen et al., 2015; Retterer et al., 2016; Huisman et al., 2017; Parenti et al., 2017; Saikusa et al., 2018; Xiao et al., 2018; Iwama et al., 2019; Kruszka et al., 2019; Downie et al., 2020; Goel and Parasivam, 2020).

Supported by these findings, a new class of disorders, named chromatinopathies, has started to emerge. Chromatinopathies are caused by variants in proteins responsible for chromatin remodeling and transcriptional regulation. The resulting global gene expression dysregulation favors the onset of a series of clinical features such as developmental delay, intellectual disability, and behavioral disturbances. CdLS, CSS, RSTS, WDSTS, and KBGS all fall within this growing family of disorders.

In this review, we aim to provide a systematic overview of the current literature to summarize all mutations in non-cohesin genes identified in association with CdLS phenotypes. For this purpose, we will discuss the functions of the affected genes, the type of variants, and the clinical features observed. By this, we will acknowledge the role of CdLS as paradigm of chromatinopathies.

### Non-canonical Cornelia de Lange Syndrome-Causing Variants

Numerous CdLS patients have been reported to carry mutations in chromatin remodelers and transcriptional regulators other than cohesin. Table 1 summarizes the described variants and provides information on the coordinates, origin and zygosity of the variants as well as gender and phenotypic CdLS scores of the individuals. Scores in parenthesis were calculated based on the published clinical features. A detailed list of the clinical features of each individual is available in Supplementary Table 1. For the purpose of this review, only individuals with a monogenic molecular diagnosis were considered. Individuals with multiple molecular diagnoses or gross deletions/insertions encompassing multiple genes were not included.

**TABLE 1:** Summary of variants in non-cohesin genes identified in CdLS-patients.

Gene	Citation	Number of patients	Patient ID	Variant type	Variant coordinates	Zygosity	Variant classification	Gender	Score	Origin	Analysis performed
ANKRD11 (NM _013275.6)	Ansari et al., 2014	3	NA	Frameshift deletion	c.6210 _6211del; p.(Lys2070Asnfs*31)	Heterozygous	Pathogenic	f	NA	*De novo*	Exome sequencing
			NA	Frameshift deletion	c.2627delT; p.(Leu876Profs*6)	Heterozygous	Pathogenic	f	NA	*De novo*	Sanger sequencing
			Decipher DDD-EDB257747	Intragenic deletion	60 kb intragenic deletion spanning exons 4–10 (chr16:89,351,798–89,412,086; hg19)	Heterozygous	Pathogenic	m	NA	*De novo*	Array-CGH
	Parenti et al., 2016	2	Patient A	Non-sense	c.5483C > A; p.(Ser1828*)	Mosaic (30% on blood DNA and 50% on fibroblast DNA)	Pathogenic	f	(12)	*De novo*	Exome sequencing
			Patient B	Frameshift deletion	c.2297 _2300delAGAA; p.(Lys766Argfs*10)	Heterozygous	Pathogenic	m	(10)	*De novo*	Exome sequencing
	Aoi et al., 2019	2	Patient 21	Frameshift deletion	c.3255 _3256del; p.(Lys1086Glufs*15)	Heterozygous	Pathogenic	f	8	*De novo*	Exome sequencing
			Patient 43	Non-sense	c.5434C > T; p.(Gln1812*)	Heterozygous	Pathogenic	m	9	*De novo*	Exome sequencing
	Cucco et al., 2020	1	Patient B	Frameshift deletion	c.3224 _3227del; p.(Glu1075Glyfs*242)	Heterozygous	Pathogenic	m	10	*De novo*	Exome sequencing
	Parenti et al., 2021	8	Patient 2	Frameshift deletion	c.1711 _1723del; p.(Thr571Alafs*15)	Heterozygous	Pathogenic	m	(9)	NA	Gene panel
			Patient 3	Non-sense	c.1977C > A; p.(Tyr659*)	Heterozygous	Pathogenic	f	(13)	NA	Gene panel
			Patient 4	Frameshift deletion	c.2398 _2401delGAAA; p.(Glu800Asnfs*62)	Heterozygous	Likely pathogenic	f	(10)	Inherited (mother)	Gene panel
			Patient 5	Frameshift deletion	c.2408 _2412del; p.(Lys803Argfs*5)	Heterozygous	Pathogenic	f	(13)	*De novo*	Gene panel
			Patient 6	Non-sense	c.2692C > T; p.(Arg898*)	Heterozygous	Pathogenic	f	(11)	*De novo*	Gene panel
			Patient 7	Frameshift duplication	c.7356dupC; p.(Lys2453Glnfs*79)	Heterozygous	Pathogenic	f	(10)	*De novo*	Gene panel
			Patient 9	Frameshift deletion	c.1903 _1907del; p.(Lys635Glnfs*26)	Heterozygous	Pathogenic	m	(8)	NA	Gene panel
			Patient 12	Splicing	c.7470 + 2T > C; p.?	Heterozygous	Likely pathogenic	m	(6)	Inherited (mother)	Gene panel
BRD4 (NM _001379291.1)	Olley et al., 2018	2	Patient 3049	Missense	c.1289A > G; p.(Tyr430Cys)	Heterozygous	Pathogenic	f	(10)	*De novo*	Gene panel
			Patient CDL038	Frameshift deletion	c.1224delinsCA; p.(Glu408Aspfs*4)	Heterozygous	Pathogenic	f	(8)	*De novo*	Gene panel
	Rentas et al., 2020	1	Patient CDL-022	Missense	c.1038G > C, p.(Lys346Asn)	Heterozygous	Uncertain significance	m	NA	Father not available. Not maternal	RNA sequencing
AFF4 (NM _014423.4)	Izumi et al., 2015	3	CHOPS T254S	Missense	c.761C > G; p.(Thr254Ser)	Heterozygous	Pathogenic	f	(3)	*De novo*	Exome sequencing
			CHOPS T254A	Missense	c.760A > G; p.(Thr254Ala)	Heterozygous	Pathogenic	m	(7)	*De novo*	Exome sequencing
			CHOPS R258W	Missense	c.772C > T; p.(Arg258Trp)	Heterozygous	Pathogenic	f	(6)	*De novo*	Exome sequencing
KMT2A (NM _001197104.2)	Yuan et al., 2015	1	CdLS-3	Non-sense	c.2233C > T; p.(Arg745*)	Heterozygous	Pathogenic	f	(13)	*De novo*	Exome sequencing
	Parenti et al., 2017	1	Patient 12	Non-sense	c.8590C > T; p.(Gln2864*)	Heterozygous	Pathogenic	m	(11)	*De novo*	Gene panel
	Aoi et al., 2019	1	Patient 27	Non-sense	c.3592C > T; p.(Gln1198*)	Heterozygous	Pathogenic	m	7	*De novo*	Exome sequencing
	Krawczynska et al., 2019	1	CdLS09	Splicing	c.4012 + 1G > A; p.?	Mosaic (48% on buccal swab DNA, 0% on blood DNA)	Likely pathogenic	m	NA	NA	Gene panel
	Demir et al., 2020	1	Case report	Frameshift deletion	c.3647 _3650delAAGA; p.(Lys1216Argfs*18)	Heterozygous	Pathogenic	f	(12)	*De novo*	Gene panel
EP300 (NM _001429.4)	Woods et al., 2014	1	Case report	Frameshift deletion	c.104 _107del; p.(Ser35Tyrfs*12)	Heterozygous	Pathogenic	m	(14)	*De novo*	Exome sequencing
	Aoi et al., 2019	1	Patient 6	In frame deletion	c.7014 _7028del; p.(His2338 _Pro2342del)	Heterozygous	Uncertain significance	f	9	Unknown	Exome sequencing
	Cucco et al., 2020	1	Patient A	Frameshift duplication	c.4408dupA; p.(Met1470Asnfs*3)	Heterozygous	Pathogenic	f	9	*De novo*	Exome sequencing
SETD5 (NM _001080517.3)	Parenti et al., 2017	2	Patient 2	Frameshift deletion	c.2212 _2213delAT; p.(Met738Valfs*27)	Heterozygous	Pathogenic	m	(9)	*De novo*	Exome sequencing
			Patient 3	Intragenic deletion	54 kb intragenic deletion spanning exons 3–19 (chr3:9,457,143-9,511,190; hg19)	Heterozygous	Pathogenic	f	NA	Father not available. Not maternal	Array-CGH
	Aoi et al., 2019	1	Patient 12	Non-sense	c.1852C > T; p.(Arg618*)	Heterozygous	Pathogenic	f	10	*De novo*	Exome sequencing
ARID1B (NM _001374828.1)	Yavarna et al., 2015	1	NA	In frame deletion	c.372 _395del; p.(Ala125 _Ser132del)	Heterozygous	Uncertain significance	NA	NA	NA	Exome sequencing
	Parenti et al., 2017	2	Patient 5	Non-sense	c.2902C > T; p.(Arg968*)	Heterozygous	Pathogenic	f	(12)	*De novo*	Exome sequencing
			Patient 6	Splicing	c.3505-2A > G; p.(Lys1169Leufs*18)	Heterozygous	Pathogenic	m	(11)	*De novo*	Gene panel
SMARCB1 (NM _003073.5)	Parenti et al., 2017	1	Patient 4	Missense	c.971A > G; p.(Lys324Arg)	Heterozygous	Uncertain significance	f	(13)	Father not available. Not maternal	Gene panel
TAF1 (NM _004606.5)	O’Rawe et al., 2015	1	Individual 4A	Missense	c.1454T > A; p.(Ile485Asn)	Hemizygous	Likely pathogenic	m	(12)	*De novo*	Exome sequencing
	Cheng et al., 2020	1	Individual 13	Missense	c.3508C > T; p.(Arg1170Cys)	Hemizygous	Likely pathogenic	m	10	*De novo*	Exome sequencing
USP7 (NM _003470.3)	Fountain et al., 2019	1	Patient 8	Intragenic deletion	31 kb intragenic deletion including a portion of 5′UTR and intron 1 and the entire exon 1 (chr16:9,085,733-9,054,621; hg19)	Heterozygous	Likely pathogenic	f	(9)	*De novo*	Genome sequencing
DDX23 (NM _004818.3)	Burns et al., 2021	2	Patient 5	Missense	c.1625G > A; p.(Arg542His)	Heterozygous	Likely pathogenic	f	(9)	*De novo*	Genome sequencing
			Patient 6	Missense	c.1583G > A; p.(Arg528His)	Heterozygous	Likely pathogenic	f	(11)	*De novo*	Genome sequencing
CSNK1G1 (NM _022048.5)	Gold et al., 2020	1	Individual 4	Missense	c.419C > T; p.(Thr140Met)	Heterozygous	Likely pathogenic	m	(9)	*De novo*	Genome sequencing
ZMYND11 (NM _001370100.5)	Aoi et al., 2019	1	Patient 53	Frameshift deletion	c.1438delG; p.(Asp480Thrfs*3)	Heterozygous	Pathogenic	m	15	Inherited (mother mosaic)	Exome sequencing
MED13L (NM _015335.5)	Aoi et al., 2019	1	Patient 5	Missense	c.6485C > A; p.(Thr2162Lys)	Heterozygous	Likely pathogenic	f	8	*De novo*	Exome sequencing
PHIP (NM _017934.7)	Aoi et al., 2019	1	Patient 56	Missense	c.1156G > A; p.(Asp386Asn)	Heterozygous	Likely pathogenic	m	6	*De novo*	Exome sequencing
TAF6 (NM _001190415.2)	Yuan et al., 2015	1	CdLS-4	Missense	c.247C > T; p.(Arg83Cys)	Homozygous	Likely pathogenic	m	(11)	Parents heterozygous	Exome sequencing
	Tuc et al., 2020	1	Individual VI-8	Missense	c.323T > C; p.(Ile108Thr)	Homozygous	Likely pathogenic	m	4	Parents heterozygous	Exome sequencing
NAA50 (NM _025146.4)	Aoi et al., 2019	1	Patient 19	Non-sense	c.93C > G; p.(Tyr31*)	Heterozygous	Likely pathogenic	m	12	*De novo*	Exome sequencing
CREBBP (NM _004380.3)	Tang et al., 2019	1	Patient 3	Frameshift deletion	c.1715delG; p.(Gly572Glufs*17)	Heterozygous	Pathogenic	f	9	*De novo*	Exome sequencing
PDGFRB (NM _002609.4)	Yavarna et al., 2015	1	NA	Missense	c.1113C > G; p.(Asn371Lys)	Heterozygous	Uncertain significance	NA	NA	NA	Exome sequencing

Many variants identified in CdLS individuals affect *bona fide* transcriptional regulators such as ANKRD11, AFF4, BRD4, SETD5, TAF1, TAF6, ZMYND11, PHIP, and MED13L.

ANKRD11 regulates gene expression through the interaction with histone-modifying proteins (Zhang et al., 2007; Li et al., 2008). Variants affecting the *ANKRD11* gene were formerly associated with KBGS (Sirmaci et al., 2011). To date, 16 individuals who received a clinical diagnosis of CdLS during infancy were found to harbor loss-of-function variants in *ANKRD11* (Ansari et al., 2014; Parenti et al., 2016, 2021; Aoi et al., 2019; Cucco et al., 2020). Clinical scores could be assessed for 13 of these 16 individuals. With an average score of 10, variants in *ANKRD11* appear to be associated with non-classic CdLS phenotypes. The relatively high frequency of *ANKRD11* variants in CdLS cohorts has motivated the inclusion of *ANKRD11* among the CdLS-genes (Kline et al., 2018).

BRD4 binds to super-enhancers elements and promotes the release of the paused RNA polymerase II (Olley et al., 2018). Three CdLS individuals with two missense substitutions and a frameshift deletion-insertion affecting *BRD4* were so far described (Olley et al., 2018). Clinical scores of 8 and 10 could be calculated for two of the three patients, thus indicating a partial overlap with CdLS.

Loss-of-function variants in *SETD5* had been initially reported in patients with moderate-to-severe intellectual disability (OMIM, #615761) (Grozeva et al., 2014). Recently, *SETD5* has been recognized as one of the most frequently mutated genes in the context of neurodevelopmental disorders (Deciphering Developmental Disorders Study, 2017; Kaplanis et al., 2020). The resulting protein carries out its function as transcriptional regulator upon interaction with two protein complexes, namely an HDAC3-containing chromatin remodeler known as Nuclear Receptor Co-Repressor (NCoR) and the RNA polymerase II-interacting complex known as Polymerase-Associated Factor 1 Complex (PAF1C) (Osipovich et al., 2016; Deliu et al., 2018). A total of three individuals carrying *SETD5* variants were identified in two independent CdLS cohorts (Parenti et al., 2017; Aoi et al., 2019). The resulting clinical scores (9 and 10) suggest a non-classic form of CdLS.

TAF1 and TAF6 are both subunits of Transcription Factor II D (TFIID), a megadalton-sized protein complex that promotes transcriptional initiation (Bieniossek et al., 2013). Variants affecting *TAF1* and *TAF6* are, respectively, associated with X-linked recessive intellectual disability (OMIM #300966) and autosomal recessive Alazami-Yuan syndrome (OMIM #617126) (Alazami et al., 2015; O’Rawe et al., 2015). Hemizygous missense substitutions in *TAF1* were identified in two individuals with CdLS (clinical scores 12 and 10), whereas two individuals were found to carry homozygous missense variants in *TAF6* (clinical scores 11 and 4) (O’Rawe et al., 2015; Yuan et al., 2015; Cheng et al., 2020; Tuc et al., 2020).

*ZMYND11*, *PHIP*, and *MED13L* were each found mutated in a single CdLS individual (Aoi et al., 2019). *ZMYND11* was the only non-cohesin-related gene altered in an individual with a clinical score of 15 and presenting with oligodactyly (Aoi et al., 2019). Prior to this discovery, *ZMYND11* had been associated with intellectual disability and behavioral disturbances (OMIM #616083); furthermore, it appears to be a critical gene in the context of the 10p15.3 microdeletion syndrome (Coe et al., 2014). The resulting protein specifically binds to trimethylated lysine 36 of histone H3 to modulate elongation of RNA polymerase II (Wen et al., 2014). *PHIP* encodes for a DNA-binding protein that localizes at promoters and transcriptional *cis-*regulatory elements (Aoi et al., 2019). Variants in *PHIP* are responsible for the obesity-associated neurodevelopmental syndrome known as Chung-Jansen syndrome (OMIM #617991) (de Ligt et al., 2012; Jansen et al., 2018). Variants in *MED13L*, a subunit of the transcriptional regulator known as Mediator complex, are instead responsible for a form of intellectual disability with dysmorphic features (OMIM #616789). Missense substitutions in *MED13L* and *PHIP* were described in two patients with CdLS-like phenotypes (clinical scores 8 and 6, respectively) (Aoi et al., 2019).

In addition, missense substitutions in AFF4, a subunit of the super elongation complex which coordinates pausing of RNA polymerase II, were identified in individuals with CHOPS (cognitive impairment, coarse facies, heart defects, obesity, pulmonary involvement, short stature, and skeletal dysplasia; OMIM #616368), who were initially suspected of having CdLS (Izumi et al., 2015). The low clinical scores of these individuals (3, 7, and 6) suggest a limited phenotypical overlap with CdLS.

Proteins that have an impact on chromatin conformation are also occasionally altered in CdLS individuals. The list of chromatin remodelers associated with CdLS comprises KMT2A, ARID1B, SMARCB1, CREBBP, and EP300.

KMT2A is a histone methyltransferase whose mutations are responsible for the onset of WDSTS (Jones et al., 2012). Five loss-of-function variants affecting *KMT2A* were reported in CdLS individuals (Yuan et al., 2015; Parenti et al., 2017; Aoi et al., 2019; Krawczynska et al., 2019; Demir et al., 2020). Clinical scores could be assessed for four of the five individuals. A score equal to or higher than 11 was calculated for three of these individuals, suggesting that *KMT2A* might be contemplated in the future as additional CdLS-gene.

ARID1B and SMARCB1 are structural components of the multisubunit protein complex named SWItch/Sucrose Non-Fermentable complex (SWI/SNF), which is known for its role as ATP-dependent chromatin remodeler (Kassabov et al., 2003). Mutations in ARID1B, SMARCB1, and other subunits of the SWI/SNF remodeler cause CSS (Santen et al., 2012; Tsurusaki et al., 2012). To date, three CdLS individuals were found to carry loss-of-function variants in *ARID1B* and one individual carried a missense substitution in *SMARCB1* (Yavarna et al., 2015; Parenti et al., 2017). Similar to *KMT2A*, the clinical scores of these patients fell within the range of classic manifestation of CdLS.

CREBBP and EP300 are part of a coactivator family characterized by intrinsic ability to acetylate histone as well as non-histone proteins and to interact with core transcription factors (Vo and Goodman, 2001; Jin et al., 2011). Mutations in *CREBBP* and *EP300* result in distinct subtypes of RSTS (Petrif et al., 1995; Roelfsema et al., 2005). In CdLS cohorts, exome sequencing led to the identification of three loss-of-function mutations in *EP300* and one out-of-frame deletion in *CREBBP* (Woods et al., 2014; Aoi et al., 2019; Tang et al., 2019; Cucco et al., 2020). With the exception of a single patient presenting with classic CdLS (Woods et al., 2014), the other individuals with variants in *CREBBP* and *EP300* appear to be associated with a rather non-classic form of CdLS (average clinical score of 9) (Aoi et al., 2019; Tang et al., 2019; Cucco et al., 2020).

The remaining CdLS-associated proteins USP7, DDX23, CSNK1G1, NAA50, and PDGFRB act indirectly on nuclear processes through their interaction with several proteins involved in genomic stability, transcriptional regulation, and chromatin remodeling.

DDX23 is a RNA helicase with a role in RNA splicing and maintenance of genomic stability through suppression of incorrect R-loops formed during transcription (Mathew et al., 2008; Sridhara et al., 2017). Two out of the nine recently published individuals with *DDX23*-related neurodevelopmental disorders presented with clinical features suggestive of CdLS and clinical scores of 9 and 11 (Burns et al., 2021).

USP7 is a deubiquitinating proteolytic enzyme with a variety of targets, including DNMT1 and members of the Polycomb multiprotein complex. By preventing their ubiquitin-dependent degradation, it promotes DNA methylation and chromatin remodeling (Maertens et al., 2010; Felle et al., 2011). Variants in *USP7* are responsible for a neurodevelopmental disorder with speech delay, altered behavior, and neurologic anomalies (Hao-Fountain syndrome, OMIM #616863) (Hao et al., 2015; Fountain et al., 2019). An individual with a CdLS score of 9 was found to carry an intragenic deletion affecting the 5′UTR and exon 1 of *USP7* (Fountain et al., 2019).

A missense substitution in *NAA50* was identified in an individual with classic CdLS (clinical score 12). NAA50 interacts with the highly conserved NatA complex composed of NAA10 and NAA15 to form the NatE complex (Deng et al., 2019; Armbruster et al., 2020). The main function of these proteins is to carry out N-terminal acetylation, a major post-translational modification to which 70–90% of proteins are subject in humans (Reddi et al., 2016; Gottlieb and Marmorstein, 2018; Deng et al., 2019). Strikingly, individuals with *NAA10* variants often show phenotypes reminiscent of CdLS (Saunier et al., 2016).

CSNK1G1 and PDGFRB possess intrinsic kinase activity through which they regulate several cellular processes including signal transduction, cell migration, and proliferation (Mori et al., 1993; Li et al., 2015). The corresponding genes have been associated with two distinct forms of syndromic neurodevelopmental disorder (Foster et al., 2020; Gold et al., 2020). Missense substitutions of each gene were identified in single individuals with CdLS-overlapping phenotypes (Yavarna et al., 2015; Gold et al., 2020).

In view of the high CdLS scores reported, *KMT2A* and the subunits of the SWI/SNF complex can be included within the extended list of CdLS genes. Variants in *ANKRD11*, *SETD5*, *EP300, CREBBP*, *BRD4*, and *TAF1* can similarly result in non-classic forms of CdLS. For this reason, these genes should be taken into account for the molecular diagnostic pipeline of CdLS. Individuals with *AFF4* variants instead present with a distinct phenotype that is only minimally overlapping with CdLS. The contribution of the other genes presented in this review in the context of CdLS still remains to be assessed (*USP7*, *TAF6*, *DDX23*, *CSNK1G1*, *ZMYND11*, *MED13L*, *PHIP*, *NAA50*, and *PDGFRB*).

### The Chromatinopathies Protein Network

**FIGURE 1:**
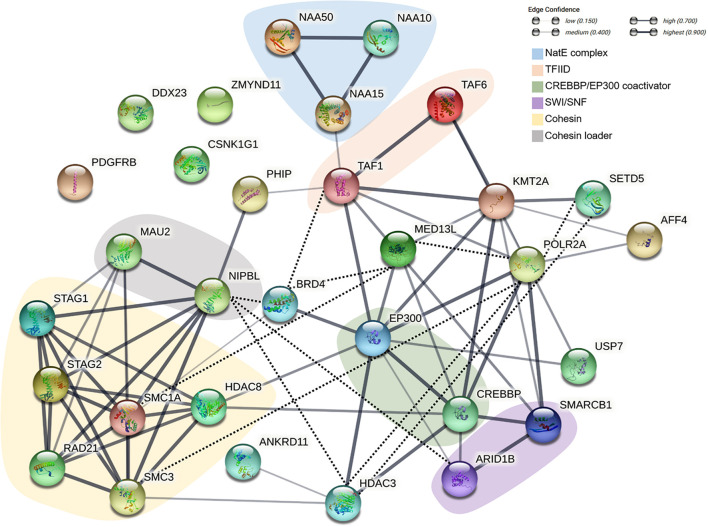
Schematic representation of the functional and physical interactions of the chromatinopathies protein network. The network was generated with the String Database (v. 11.5) (Szklarczyk et al., 2019). Empty nodes represent proteins of unknown 3D structure, while filled nodes indicate proteins with known or predicted protein structure. Line thickness indicates the strength of data support. Interactions were established based on co-expression or data from either curated databases or experimentally determined. The network was subsequently manually curated (dotted black line) based on more recent literature.

Cohesin and non-cohesin proteins involved in the pathogenesis of CdLS and other neurodevelopmental disorders do not only share overlapping functions. These proteins are profoundly interconnected and give rise to a genuine chromatinopathies protein network. Figure 1 provides a schematic overview of the network; here, the chromatinopathies proteins are illustrated in light of their physical and functional interactions. Central nodes of the network such as HDAC3 or POLR2A, despite not being associated with CdLS so far, are depicted to allow a more comprehensive outlook of the network. It is apparent how the proteins involved act concertedly and regulate each other with the aim of controlling transcription. The tightly regulated interplay of components is in fact responsible for the coordinated expression of numerous genes. Given the major role of RNA polymerase II, mediator, and TFIID complexes in the context of gene expression regulation, it is not surprising that several chromatinopathies proteins either interact with or indirectly control the levels or activity of these three main effectors. For instance, the canonical CdLS-protein complex, i.e., cohesin, can directly influence the amount of RNA polymerase II available at the promoters of several genes (Schaaf et al., 2013). Furthermore, cohesin functionally and physically interacts with the mediator complex to connect enhancers and promoters of active genes (Kagey et al., 2010). The recruitment of RNA polymerase II is also dependent on HDAC3 (Wang et al., 2018), a histone deacetylase that equally appears to be one of the central nodes of the chromatinopathies network. The roles of HDAC3 within the network are in fact plentiful, as it was reported to interact directly with numerous players with the aim of “fine-tuning” transcription. The HDAC3-interacting proteins comprise SETD5, ANKRD11, EP300, CREBBP, and the cohesin loader NIPBL (Zhang et al., 2004; Jahnke et al., 2008; Sankar et al., 2008; Osipovich et al., 2016; Deliu et al., 2018). Remarkably, whereas mutations affecting RNA polymerase II have already been associated with a neurodevelopmental disorder that overlaps with chromatinopathies (OMIM, #618603) (Haijes et al., 2019), variants in *HDAC3* have never been reported. Taking into account the central role of HDAC3 in the transcription process, a possible identification of disease-causing *HDAC3* variants can be envisaged.

Following its recruitment to the DNA, the dynamics and activity of RNA polymerase II are further subject to regulation through proteins like SETD5 and BRD4 (Osipovich et al., 2016; Lee et al., 2017; Deliu et al., 2018). Specifically, BRD4 can control transcription by promoting the enrichment of RNA polymerase II, mediator and TFIID at target genes (Lee et al., 2017) and through its interaction with NIPBL and different cohesin subunits (Olley et al., 2018). In turn, the acetyltransferase EP300 and CREBBP seem to be responsible for BRD4 recruitment to enhancers (Lee et al., 2017). Additional data suggest that EP300 and CREBBP contribute to chromatin architecture along with the mediator complex (Zhang et al., 2020), the methyltransferase KMT2A (Goto et al., 2002), and the SWI/SNF complex (Alver et al., 2017). The latter is itself responsible for the recruitment of the cohesin loader to nucleosome-free regions (Lopez-Serra et al., 2014) and is as well able to interact with RNA polymerase II and the TFIID complex (Sharma et al., 2003).

This is certainly a simplistic view of the incredibly complex and perfectly orchestrated process that is transcription, but conveys the idea of how much interconnected the chromatinopathies protein network is. The level of synergy of the network is so high that variants of a single factor will inevitably result in an altered function of the other players.

## Conclusion

Several proteins with interdependent roles belong to the chromatinopathies protein network. Disease-causing variants in the corresponding genes are accountable for the onset of distinct but overlapping neurodevelopmental disorders, of which CdLS is a paradigm. Whether or not the resulting transcriptional dysregulation converge on a common pathway or set of genes is an intriguing possibility that is worth exploring for therapeutic purposes.

## Author Contributions

Both authors contributed to the manuscript drafting, read and approved the submitted version.

## Conflict of Interest

The authors declare that the research was conducted in the absence of any commercial or financial relationships that could be construed as a potential conflict of interest.

## Publisher’s Note

All claims expressed in this article are solely those of the authors and do not necessarily represent those of their affiliated organizations, or those of the publisher, the editors and the reviewers. Any product that may be evaluated in this article, or claim that may be made by its manufacturer, is not guaranteed or endorsed by the publisher.
